# Risk of second bone sarcoma following childhood cancer: role of radiation therapy treatment

**DOI:** 10.1007/s00411-013-0510-9

**Published:** 2014-01-14

**Authors:** Boris Schwartz, Mohamed Amine Benadjaoud, Enora Cléro, Nadia Haddy, Chiraz El-Fayech, Catherine Guibout, Cécile Teinturier, Odile Oberlin, Cristina Veres, Hélène Pacquement, Martine Munzer, Tan Dat N’Guyen, Pierre-Yves Bondiau, Delphine Berchery, Anne Laprie, Mike Hawkins, David Winter, Dimitri Lefkopoulos, Jean Chavaudra, Carole Rubino, Ibrahima Diallo, Jacques Bénichou, Florent de Vathaire

**Affiliations:** 1 Radiation Epidemiology Group, Unit 1018 INSERM, Institut Gustave Roussy, Rue Camille Desmoulins, 94805 Villejuif, France; 2 Institut Gustave Roussy, 94805 Villejuif, France; 3 Université Paris-Sud, 94800 Villejuif, France; 4 Institut Curie, Paris, 75005 Paris, France; 5 Institut Jean Godinot, 51092 Reims, France; 6 Centre Antoine Lacassagne, 06100 Nice, France; 7 Centre Claudius Régaud, 31300 Toulouse, France; 8 Department of Public Health and Epidemiology, Centre for Childhood Cancer Survivor Studies, University of Birmingham, Birmingham, UK; 9 INSERM, U657, 76031 Rouen, France; 10 Unité de Biostatistique, Centre Hospitalier Universitaire (CHU) de Rouen, 76031 Rouen, France; 11 Université de Rouen, 76031 Rouen, France

**Keywords:** Bone sarcoma, Childhood cancer, Iatrogenous effects, Radiation therapy, Secondary tumor

## Abstract

Bone sarcoma as a second malignancy is rare but highly fatal. The present knowledge about radiation-absorbed organ dose–response is insufficient to predict the risks induced by radiation therapy techniques. The objective of the present study was to assess the treatment-induced risk for bone sarcoma following a childhood cancer and particularly the related risk of radiotherapy. Therefore, a retrospective cohort of 4,171 survivors of a solid childhood cancer treated between 1942 and 1986 in France and Britain has been followed prospectively. We collected detailed information on treatments received during childhood cancer. Additionally, an innovative methodology has been developed to evaluate the dose–response relationship between bone sarcoma and radiation dose throughout this cohort. The median follow-up was 26 years, and 39 patients had developed bone sarcoma. It was found that the overall incidence was 45-fold higher [standardized incidence ratio 44.8, 95 % confidence interval (CI) 31.0–59.8] than expected from the general population, and the absolute excess risk was 35.1 per 100,000 person-years (95 % CI 24.0–47.1). The risk of bone sarcoma increased slowly up to a cumulative radiation organ absorbed dose of 15 Gy [hazard ratio (HR) = 8.2, 95 % CI 1.6–42.9] and then strongly increased for higher radiation doses (HR for 30 Gy or more 117.9, 95 % CI 36.5–380.6), compared with patients not treated with radiotherapy. A linear model with an excess relative risk per Gy of 1.77 (95 % CI 0.6213–5.935) provided a close fit to the data. These findings have important therapeutic implications: Lowering the radiation dose to the bones should reduce the incidence of secondary bone sarcomas. Other therapeutic solutions should be preferred to radiotherapy in bone sarcoma-sensitive areas.

## Introduction

Excess of incidence and mortality from a second malignant neoplasm (SMN) is an increasing concern among survivors of childhood cancers (Reulen et al. 2011; Olsen et al. 2009; Friedman et al. 2010; Bassal et al. 2006). Despite their rarity, sarcomas accounted for 19 % of the SMN among survivors aged less than 15 years, 10 % among those aged 15–39 years, 5 % among those aged 40–59 years and almost 0 % for older ages (Olsen et al. 2009). Moreover, bone sarcoma exhibits the highest overall standardized incidence ratio (SIR) for any SMN category (Reulen et al. 2011). However, although radiation dose has been demonstrated to be a major risk factor, the role of radiation dose in the risk of secondary bone sarcoma among survivors of childhood cancers is currently unclear. Indeed, case–control studies investigating the relation between the radiation dose at a particular bone site and the risk of developing bone sarcoma at this site have reached widely different conclusions (Berrington de Gonzalez et al. 2012; Henderson et al. 2012; Kleinerman et al. 2005; Le Vu et al. 1998; Hawkins et al. 1996; Wong et al. 1997; Tucker et al. 1987). This may be due to insufficient follow-up or because of the design of the case–control studies in which evaluation of the dose–response relationship requires the use of the “local dose” of radiation at the sarcoma site of the case and at the same site for its matched controls. When nested in a cohort, practical constraints related to control selection may induce reduction in the case–control study sample and therefore cause some biases.

The highlights of the current study, in addition to being based on a valuable dataset with a long follow-up, are that detailed information on radiotherapy was available and estimates of radiation dose at many sites in each bone were performed for each subject; thus, one could explore the dose–response relationship in a cohort study design instead of through the usual case–control analysis design, taking full advantage of the available data.

The purposes of this cohort study were (1) to investigate the role of the radiation dose in the risk of secondary bone sarcoma in survivors of childhood cancer; (2) to identify primary neoplasm types that contribute most to the risk of secondary bone sarcoma; and (3) to develop an innovative approach that allows modeling of the dose–response relationship in a cohort study design.

## Materials and methods

### Patients

#### Cohort

A cohort of 4,171 children treated in France and Britain was constituted between 1985 and 1995, comprising patients who were alive without secondary bone sarcoma 5 years after a first solid cancer diagnosis made before the year 1986 and before the ages of 16 years for French patients and 15 years for English patients, who were followed up thereafter. One hundred and forty-three patients who had an osteosarcoma as the first cancer were excluded because, in the case that they developed bone sarcoma during follow-up, it would be impossible to determine whether the bone sarcoma was a second cancer or a recurrence of the first lesion. However, patients treated for Ewing’s sarcoma in their childhood were not excluded because if they developed bone sarcoma as second cancer, one would be able to determine whether it was a recurrence or a new cancer, based on histology.

Follow-up for the occurrence of death or second cancer of the 2,967 French patients from the diagnosis of childhood cancer relied exclusively on medical records from the treatment centers and general practitioners and a self-completed questionnaire. Cancers declared from the questionnaire were required to be validated by contacting the general practitioner or checking medical records; otherwise, they were not taken into account in analyses. This questionnaire was based on that used in the British Childhood Cancer Survivor Study (Hawkins et al. 2008). A total of 1,825 patients returned the completed questionnaire (sent to 2,449 alive patients) by December 31, 2010, which is the end point of our study.

A total of 1,204 British patients were followed up for the occurrence of a second cancer and death using the National Health Service Central Registers (Office of National Statistics 2006; Hawkins and Swerdlow 1992). They were followed up until the occurrence of a bone sarcoma, their death or December 31, 2006, whichever came first.

#### Case–control sampling

To make our results more comparable to those of published case–control studies, a nested case–control analysis was also performed within the cohort. Five controls were individually matched to second cancer sarcoma cases on sex, age, year of diagnosis, type of first cancer and duration of follow-up since the first cancer diagnosis. In this analysis, a case could potentially serve as a control for a case that had occurred earlier.

### Case identification

A case was characterized by a patient having a tumor whose histology was defined as a bone sarcoma in the ICD (ICD codes 170.0–170.9). French bone sarcoma cases were identified from self-questionnaire, medical records and the National Registry of Causes of Death. English bone sarcoma cases were identified from the National Health Service Central Registers (Hawkins and Swerdlow 1992). Only bone sarcoma cases validated through a copy of the pathological record were considered as cases.

### Radiation dosimetry

Retrospective dosimetric estimation was performed by reconstructing the body, i.e., developing a mathematical phantom of the individual patient from medical data files, at the time of treatment (Francois et al. 1988a, b) and the radiation therapy circumstances for each patient. Radiation doses were estimated at 188 points, fairly spatially allocated in the human body, from which 80 were located in bones. The Dos_EG software used was developed specifically for these dose calculations (Shamsaldin et al. 1998; Diallo et al. 1996; Francois et al. 1988a, b).

In the cohort analysis, the radiation dose was estimated in 59 bones for each of the 2,879 patients who had received radiotherapy during the follow-up period, i.e., up to 2 years before the end of their follow-up, because it is believed that the iatrogenic effects of radiotherapy do not appear within 2 years of treatment. If several points for which doses have been estimated were located within the same bone (some long bones, such as the femur, are long enough that two estimation points have been allocated within them), the mean dose was attributed to obtain a single dose by bone. In this case, the bone dose was more representative of the mean bone radiation dose than if the dose was estimated just based on one point into the bone. Obviously, if we could have estimated hundreds of doses in each bone, the accuracy of the average bone dose would have been much better, but unfortunately, we did not have these data. However, we do not expect it would affect the risk estimates significantly.

In the case–control study, only the local dose of radiation was considered, which means that for each case, the dose to the bone sarcoma site has been estimated, and for each matched control, the dose to the same site has also been estimated.

No dose estimation was performed for patients treated with brachytherapy because the focus was only on external radiotherapy here. Hence, five cases were excluded from the dose–response relationship estimation for this reason.

### Bones

**Fig. 1:**
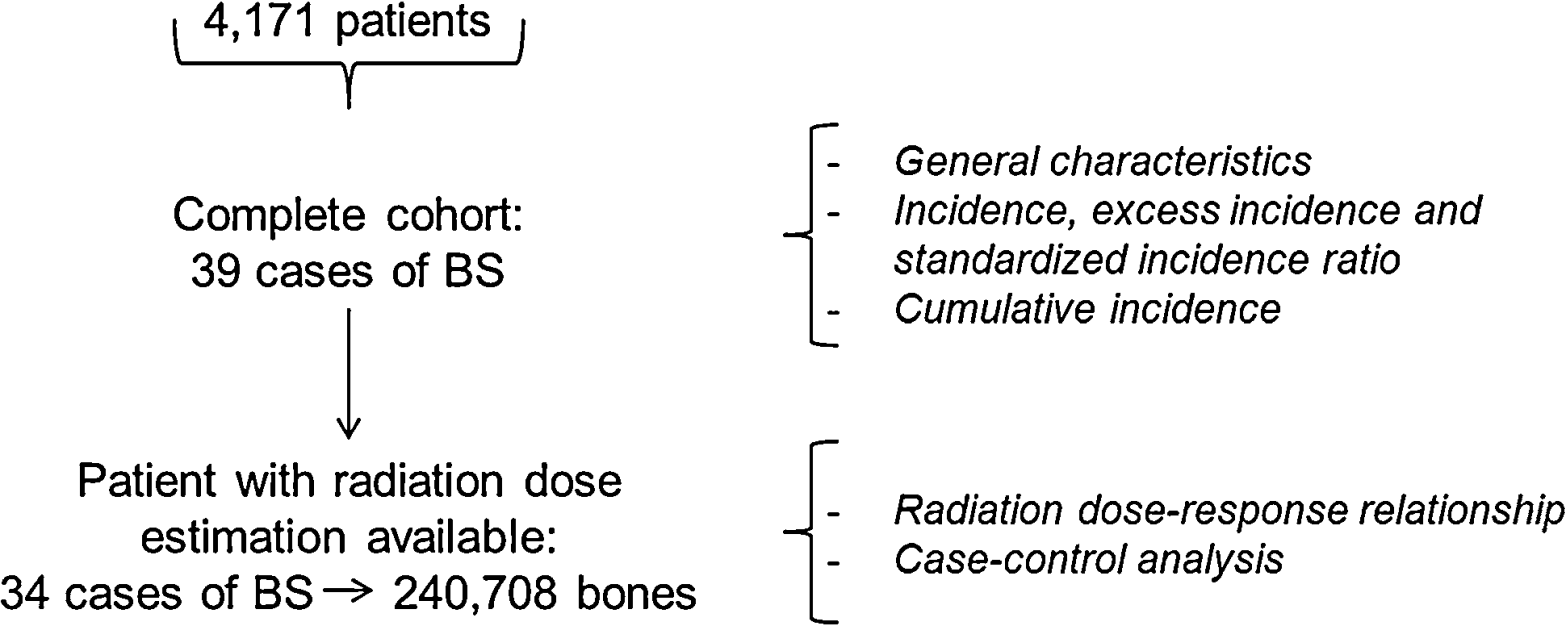
Study population according to analyses; *BS* bone sarcoma

**Fig. 2:**
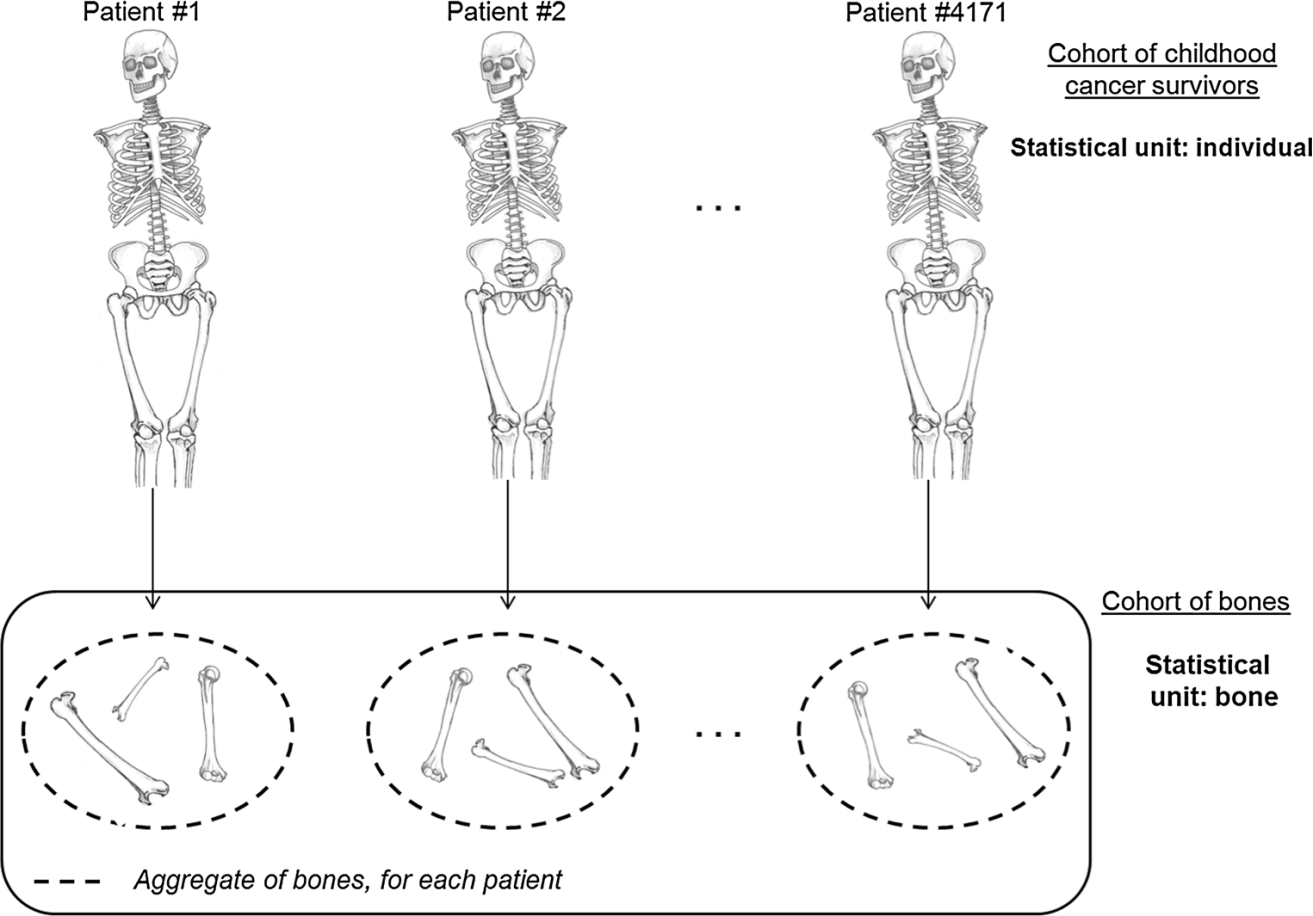
Construction of the cohort of bones

To take into account the wide heterogeneity of the radiation dose delivered throughout the body during radiation therapy, the radiation dose received at the bone level was considered here. The average human adult skeleton consists of 206 bones (Ramé and Thérond 2006). Bones of the arms, hands and feet (altogether 112 bones) were not considered because of the lack of precision concerning the position of the patient during the radiotherapy courses (two cases excluded, Fig. 1). Apart from these bones, there was missing information on several small bones of the face and mini-bones (a total of 23 bones). However, all the 71 remaining bones, i.e., the largest in terms of volume, had radiation dose estimated at least at one point and were thus all taken into account. The body of each patient was divided into 59 bones (ribs were grouped in pairs; Fig. 2).

### Chemotherapy quantification

Drugs were grouped into seven classes according to their known mechanisms of action in the cell: vinca alkaloids, antimetabolites, alkylating agents, anthracyclines, cytotoxic antibiotics, epipodophyllotoxins and other drugs. The total cumulative amount of drugs administered for each class was expressed in moles per square meter, but also calculated in milligrams per square meter, as usual. Doses were expressed per square meter to take account of the body surface area that was used for dose administration.

### Statistical methods

The SIRs of bone sarcoma were estimated as the ratio of observed to expected overall numbers of incident cases of bone sarcoma. The expected number of incident cases of bone sarcoma was defined as the number of person-years of follow-up in the given cell multiplied by the corresponding incidence rate of bone sarcoma from the British national cancer incidence rates (Office of National Statistics 2006). British rates were used for patients both in France and in Britain because no reference incidence rate estimates of bone sarcoma were available in France. Inference for the SIR relied on exact calculations based on the Poisson distribution of the observed number of events (Belot et al. 2008). The absolute excess risk (AER) was estimated as the difference between the observed and expected number of incident bone sarcomas, divided by the number of person-years of follow-up.

Each bone of the same child could have received very different radiation doses during radiotherapy courses. We therefore performed analyses of the relationship between the radiation doses received and the risk of bone sarcoma *using the bone as the statistical unit*. In this analysis, each patient represented 59 bones, except for the 99 patients who had had a leg amputated, for whom only their remaining bones were taken into account. This method requires that every bone of each patient from the cohort is accounted for (if not amputated), in order to allow comparisons between the same bones of different patients.

As the dynamics of bone growth and radiation sensitivity were likely to be more similar among bones of the same structure and in the same area than between those in various areas of the body, six skeleton parts were defined within the human body in order to run a parsimonious stratified analysis: the head, the pectoral girdle, the ribs, the spine, the pelvis and the legs. Thanks to this type of analysis, bones were compared with other bones of the same area of the skeleton and this allowed us to take into account radiosensitivity heterogeneity in the whole body. In other words, this method did not allow for reducing radiation dose heterogeneity, but for better considering it and reducing variance of risk estimates.

An internal analysis was conducted using Cox’s proportional hazard regression model for clustered data, in order to account for the lack of independence between the bones of the same patient, and with stratification on skeleton parts. The marginal approach of Wei et al. (1989), using robust “sandwich” variance estimates, was used. The Fine and Gray method with multiple imputations was used to take into account death as a competing risk (Pintilie 2011; Fine and Gray 1999; Ruan and Gray 2008; Resche-Rigon et al. 2006; Gooley et al. 1999). Therefore, the cumulative incidence and its variance were calculated based on the Marubini and Valsecchi formula (Marubini and Valsecchi 1995, p. 341, equation 10.12). In these models, the timescale was attained age, and entry time was the age at diagnosis of the first cancer plus 2 years for French and 5 years for English patients (Thiebaut and Benichou 2004).

In order to evaluate the dose–response relationship between the bone radiation dose and the risk of bone sarcoma, the following models were fitted within Cox regression (Breslow and Day 1987):

1. Basic: hazard ratio = 1
2. Linear: hazard ratio = 1 + *β*_1_dose
3. Linear quadratic: hazard ratio = 1 + *β*_1_dose + *β*_2_dose^2^
4. Linear exponential: hazard ratio = (1 + *β*_1_dose) × e^*γ*dose^
5. Linear quadratic exponential: hazard ratio = (1 + *β*_1_dose + *β*_1_dose^2^) × e^*γ*dose^

with dose denoting the radiation dose to the bone.

Nested models were compared using the likelihood ratio test, and confidence intervals (CIs) for model parameters were estimated using profile likelihood (Moolgavkar and Venzon 1987). Only results derived from the selected model have been reported in this paper.

Due to the small number of incident bone sarcoma cases in each skeleton part (16 cases at most), a separate analysis for each set would have been underpowered. Consequently, only overall analyses of the whole skeleton, with stratification on the skeleton parts, were performed.

In the case–control analysis, conditional logistic regression was used. The adjustment variables were the same as in the cohort analysis, namely gender, age at diagnosis of the first cancer, type of first cancer, chemotherapy administration, number of drugs and spinal dose of radiation.

The EPICURE statistical software (Preston et al. 1993) and SAS^®^ 9.3 (SAS Inst. Inc., Cary, NC) were used for the analyses. All tests were two-sided, and a *p* value below 0.05 was considered significant. The proportional hazard assumption was verified in the final model.

## Results

### Cohort

The median follow-up of 4,171 patients was 26 years following the diagnosis of the first cancer (Table 1). From 5 to 37 years of age, 39 patients had developed bone sarcoma in 16 different bones. The majority of bone sarcomas developed in the legs (12) or head (11). Most of these cases had developed osteosarcoma (30); five had developed chondrosarcoma; and the four other cases had developed histiocytofibroma (2), Ewing’s sarcoma and sarcoma without further precision. Of these cases, 27 were men and 12 women (*p* = 0.08). Among the 2,879 patients who had received radiotherapy during the follow-up period, 33 had developed bone sarcoma. The median absorbed dose of radiation to the bones was 0.48 Gy (range 0.00–179.83 Gy).

**Table 1:** General characteristics of 4,171 survivors of childhood cancer

Country	France	Britain
No. of patients (%)	2,967 (71.1)	1,204 (28.9)
Years of treatment: median (range)	1977 (1946–1985)	1974 (1942–1985)
Age at diagnosis in years: median (range)	4 (0–20)	5.0 (0–15)
Follow-up in years: median (range)	26 (5–61)	28 (5–62)
Sex: no. (%) of males/no. (%) of females	1,626 (54.8)/1,341 (45.2)	675 (56.1)/529 (43.9)
First cancer treatment no. (%)		
Neither CT nor RT	212 (7.1)	196 (16.3)
CT but no RT	681 (23.0)	203 (16.9)
RT but no CT	578 (19.5)	363 (30.1)
RT and CT	1,496 (50.4)	442 (36.7)
Death before end of study no. (%)	561 (18.9)	233 (19.4)
Bone sarcomas during follow-up no. (%)	35 (1.2)	4 (0.3)
Radiation dose^a^ (Gy): median (range)		
Head	0.5 (0–110)	0.5 (0–126)
Pectoral girdle	0.7 (0–180)	0.6 (0–90)
Ribs	1.0 (0–99)	0.6 (0–90)
Spine	1.1 (0–111)	0.6 (0–99)
Pelvis	0.8 (0–47)	0.3 (0–61)
Legs	0.1 (0–81)	0.1 (0–96)

*CT* chemotherapy, *RT* radiotherapy, but no brachytherapy

^a^For irradiated patients only, except for those treated by brachytherapy. Not available for arms

The cumulative incidences of bone sarcoma at 10, 20 and 35 years of age were, respectively, 0.3 % (95 % CI 0.1–0.4 %), 0.9 % (95 % CI 0.6–1.2 %) and 1.1 % (95 % CI 0.8–1.5 %). The overall incidence was about 45-fold higher than expected in the general population (95 % CI 31.0–59.8) (Table 2).

**Table 2:** Incidence of bone sarcoma, excess incidence and standardized incidence ratio

	Patients still followed up	Bone sarcoma				
		# Observed cases	# Expected cases^a^	Annual incidence per 10^5^ person-years	AER per 10^5^ person-years^b^	SIR^c^
Total	4,171	39	0.87	35.9 (24.8–47.9)	35.1 (24.0–47.1)	44.8 (31.0–59.8)
Years after diagnosis						
5–9	4,171	14	0.27	42.8 (21.4–67.2)	41.9 (20.6–66.4)	51.9 (25.9–81.5)
10–19	3,867	21	0.37	56.8 (35.2–81.1)	55.8 (34.1–80.1)	56.8 (35.1–81.1)
20–29	3,388	3	0.15	11.7 (0.0–27.4)	11.1 (0.0–26.8)	20.0 (0.0–46.7)
≥30	1,575	1	0.07	7.5 (0.0–22.4)	6.9 (−0.5 to 21.9)	14.3 (0.0–42.9)
Attained age (years)						
5–9	4,171	4	0.06	28.7 (7.2–57.5)	28.3 (6.8–57.1)	66.7 (16.7–133.3)
10–19	4,088	27	0.45	85.2 (53.6–119.9)	83.8 (52.2–118.5)	60.0 (37.8–84.4)
20–29	3,643	4	0.22	12.2 (3.1–24.4)	11.5 (2.4–23.7)	18.2 (4.5–38.4)
≥30	2,521	4	0.14	14.7 (3.7–29.3)	14.1 (3.2–28.8)	28.6 (7.1–57.1)
Age at diagnosis (years)						
0–4	2,184	22	0.47	37.7 (22.3–54.9)	36.9 (21.5–54.1)	46.8 (27.7–68.1)
5–9	1,041	11	0.22	41.1 (18.7–67.2)	40.3 (17.9–66.4)	50.0 (22.7–81.8)
≥10	946	6	0.18	25.4 (8.5–46.6)	24.7 (7.7–45.8)	33.3 (11.1–61.1)
Type of treatment						
Surgery alone	408	1	0.09	8.0 (0.0–23.9)	7.3 (−0.7 to 23.3)	11.1 (0.0–33.3)
CT but no RT	884	5	0.17	25.2 (5.0–50.4)	24.4 (4.2–49.6)	29.4 (5.9–58.8)
RT but no CT	941	5	0.21	17.3 (3.5–34.7)	16.6 (2.7–33.9)	23.8 (4.8–47.6)
RT and CT	1,938	28	0.39	59.0 (37.9–82.2)	58.2 (37.1–81.3)	71.8 (46.2–100.0)

*CT* chemotherapy, *RT* radiotherapy, but no brachytherapy

^a^From the United Kingdom general population rates

^b^ *AER* absolute excess risk, defined as [(observed − expected)/person-years]

^c^ *SIR* standardized incidence ratio, defined as (observed/expected)

The SIR stabilized then decreased with increasing attained age (Table 2), except that the SIR was higher after 30 years than between 20 and 29 years. No trend was found concerning the SIR with time since first cancer diagnosis. Also, no trend of the AER was observed with time since first cancer diagnosis, nor with increasing attained age. Both the SIR and AER were higher following radiotherapy plus chemotherapy than they were following either of these modalities alone.

In the univariate analysis, type of first cancer and risk of bone sarcoma as a secondary cancer were closely linked (Table 3, *p* < 0.001). Patients who had retinoblastoma, Ewing’s, soft tissue sarcomas and Hodgkin’s disease had a significantly higher risk of bone sarcoma than patients who had a nephroblastoma as the first cancer.

**Table 3:** Risk of bone sarcoma according to the type of first cancer

	No. of cases/no. of patients	Radiation therapy (%)	Average bone dose (Gy), mean, median (range)^a^	AER per 10^5^ PYR (95 % CI)	SIR^b^ (95 % CI)	Unadjusted HR (95 % CI)
Nephroblastoma	2/851	72.7	6.6, 5.7 (0.1–24.5)	7.6 (**−**0.8 to 20.3)	10.5 (0.0–26.3)	1 (Ref)
Neuroblastoma	2/573	55.2	5.1, 4.0 (0.1–29.1)	12.6 (**−**0.8 to 32.7)	16.7 (0.0–41.7)	1.6 (0.2–11.5)
Hodgkin’s disease	5/378	91.3	13.0, 12.6 (0.0–40.0)	51.2 (9.6–103.2)	62.5 (12.5–125.0)	6.2 (1.2–31.5)
Non-Hodgkin lymphoma	3/459	59.5	5.8, 4.8 (0.1–25.0)	27.1 (**−**0.8 to 64.3)	33.3 (0.0–77.8)	3.1 (0.5–18.4)
Soft tissue sarcoma	11/535	62.6	3.2, 1.9 (0.0–17.4)	76.6 (34.4–125.8)	100.0 (45.5–163.6)	9.3 (2.0–41.8)
Ewing’s sarcoma	6/141	92.2	3.8, 2.7 (0.0–16.8)	179.5 (59.2–329.7)	200.0 (66.7–366.7)	20.1 (4.1–98.8)
CNS tumor	1/690	83.0	8.0, 3.7 (0.3–35.0)	4.6 (**−**0.7 to 15.3)	7.1 (0.0–21.4)	0.6 (0.1–6.9)
Gonadal tumor	1/227	38.3	7.9, 7.6 (0.5–28.8)	16.7 (**−**0.7 to 51.5)	25.0 (0.0–75.0)	2.1 (0.2–22.7)
Retinoblastoma	7/144	81.3	1.8, 1.2 (0.1–26.3)	220.4 (62.3–410.0)	233.3 (66.7–433.3)	25.0 (5.2–120.9)
Other first cancers	1/173	48.6	7.5, 6.4 (0.2–28.7)	21.5 (**−**0.7 to 65.9)	33.3 (0.0–100.0)	2.7 (0.2–29.4)
Entire cohort	39/4,171	69.0	6.8, 4.7 (0.0–40.0)	35.1 (24.0–47.1	44.8 (31.0–59.8)	–

*PYR* person-years, *CI* confidence interval, *HR* hazard ratio in a Cox’s proportional hazards model with clustering in order to take into account the fact that several bones are from the same patients

^a^In patients with radiotherapy, except for those treated by brachytherapy

^b^As compared to the general British population: *AER* absolute excess risk, defined as [(obs **−** exp)/person-years], *SIR* standardized incidence ratio, defined as (obs/exp)

Bone sarcomas were more likely to occur in the pelvis (HR = 49.2, 95 % CI 15.0–161.2), legs (HR = 11.0, 95 % CI 3.9–31.1) and head (HR = 11.3, 95 % CI 3.9–32.4) than in the trunk. After adjustment on the radiation dose, hazard ratios were higher: 93.7 (95 % CI 27.2–327.6), 67.5 (95 % CI 18.3–249.1) and 12.9 (95 % CI 4.4–37.4) for pelvis, legs and head, respectively.

### Risk model for radiations

**Fig. 3:**
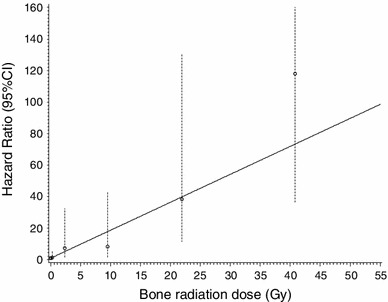
Hazard ratio for bone sarcoma according to the radiation dose to the bone; the *circles* represent observed values by radiation dose, and *vertical bars* represent corresponding 95 % CI. The* curve* is the prediction using the following model: HR = 1 + 1.773 * dose; 95 % CI for 1.773: 0.6213–5.935; six levels of dose represented are no radiation dose, 0–1, 1–5, 5–15, 15–30 Gy and more than 30 Gy. *Note* The upper-bound confidence limit of the last category of dose has been truncated for better readability

Upon modeling the association between the hazard rate of bone sarcoma and bone doses, a linear model fitted the data the most adequately (Fig. 3). The excess relative risk (ERR) per Gy in this model was 1.78 (95 % CI 0.62–5.94). This entailed the same hazard per additional Gray at low (less than 1 Gy) as at high (more than 10 Gy) doses. Compared with patients who did not receive radiation therapy, the risk of bone sarcoma for those who received more than 30 Gy was almost 120-fold higher (Table 4; Fig. 3).

**Table 4:** Bone sarcoma’s risk according to the bone radiation dose

	No radiation	0–1 Gy	1–5 Gy	5–15 Gy	15–30 Gy	30 Gy or more
Bone sarcomas/exposed bones	4/80,643	5/90,574	3/22,306	2/16,467	7/17,060	13/13,658
Median bone radiation dose^a^ (range)	0	0.2 (0.0–1.0)	2.0 (1.0–5.0)	9.2 (5.0–15.0)	21.5 (15.0–30.0)	38.1 (30.0–179.8)
HR^b^ (95 % CI)	1 (Ref)	1.4 (0.3–5.7)	7.3 (1.6–32.3)	8.2 (1.6–42.9)	38.4 (11.3–130.5)	117.9 (36.5–380.6)
Median local bone radiation dose^a^ (range)	0	0.2 (0.0–0.7)	2.4 (1.1–4.7)	8.9 (5.2–13.9)	21.8 (16.5–28.2)	42.9 (30.7–73.1)
OR^c^ (95 % CI)	1 (Ref)	2.0 (0.4–9.5)	34.7 (2.2–535.5)	22.2 (1.5–324.0)	415.5 (20.1–8,595.5)	898.0 (27.5–29,325.7)

^a^Dose in Gray (Gy)

^b^ *HR* hazard ratio in a Cox’s proportional hazards model with clustering in order to take into account the fact that several bones are from the same patients, adjustment for age at diagnosis, sex, type of first cancer, chemotherapy and spinal radiation dose, and stratification on the skeleton parts

^c^ *OR* odds ratio in a conditional logistic regression in a nested case–control analysis (34 cases/170 controls, matched on sex, age and year of diagnosis and type of the first cancer), adjustment on chemotherapy and spinal radiation dose

### Chemotherapy

Chemotherapy administration was a nonsignificant risk factor for bone sarcoma (HR = 1.9, 95 % CI 0.6–5.9) after adjustment. We failed to evidence a significant role for a given drug category, even alkylating agents.

No interaction was found between the dose of ionizing radiation and chemotherapy in the cohort (*p* = 0.20) or case–control analysis (*p* = 0.43).

### Evaluation of the approach

Odds ratios estimated from the nested case–control study were higher than hazard ratio estimates from the cohort analysis, with CI very much larger in the case–control study due to the small number of cases (Table 4). However, CI overlaps, hence there were no statistical differences between the two approaches, but that did not mean that they are similar. Nevertheless, we strongly suppose that the nonsignificance is a consequence of the lack of power due to the low number of cases and that the cohort analysis was more accurate.

## Discussion

Despite the relatively large size of the cohort and the long duration of follow-up, the main limitation of our study is the small number of bone sarcomas: 39 cases, of whom only 34 had available radiation doses.

Also, we were unable to take into account all the bones of the body and the total radiation dose received by each bone and its distribution. For example, for long bones such as the femur, the radiation dose estimation was performed only on two physical sites. Nonetheless, the radiation dose was estimated in at least one site for the majority of bones.

Several factors add credence to the results presented. For example, previous medical and treatment data were collected exhaustively. The response rate in French patients who were alive when the questionnaire was sent was 75 %, higher than in the Childhood Cancer Survivor Study (Robison et al. 2002), and the nonresponders among these patients were nevertheless followed up for 17 years on average, whereas British patients were followed up from a National Cancer Registry. Among cases that were only registered as cause of death, only the validated ones were considered as cases in the analyses. Hence, all cases in our analyses had been confirmed by histopathologists.

In our cohort, the ERR per Gy was 1.78 (95 % CI 0.62–5.94), i.e., the hazard ratio for a dose of 1 Gy was 2.8 (95 % CI 1.6–6.9). This value is lower but compatible with that expected from the last report on the analysis of Hiroshima and Nagasaki survivors in which a threshold dose of 0.85 Gy was observed, with each Gray above this dose multiplying the risk of bone sarcoma by 7.5 (95 % CI 1.3–23.1) (Samartzis et al. 2011). Our results are also compatible with those of Tucker et al. (1987), Hawkins et al. (1996) and Henderson et al. (2012), although all of them only considered the local radiation dose. Indeed, Tucker pointed out an increased risk with an increased radiation dose, with a relative risk of about 40 for doses above 60 Gy relative to no radiation. Hawkins et al. estimated a relative risk of 93.4 (95 % CI 6.8–1,285.4) for doses between 30 and 50 Gy, which is close to our estimate of 117.9 (36–5,380.6) for doses greater than 30 Gy. The same conclusions could be drawn from Henderson’s paper, which exhibits an excess odds ratio per Gray (EOR/Gy) of 1.32 (95 % CI 0.44–4.22) and an OR of 114.1 (95 % CI 13.5–964.8) for doses higher than 50 Gy. However, contrary to the findings of Hawkins et al., no decline in the risk of bone sarcoma was observed for very high doses (more than 15 Gy). These three papers also noted an increase in the risk of bone sarcoma if patients had been treated with chemotherapy, which is consistent with our results, although we did not identify a drug class specifically associated with this increase, even when considering alkylating agents. The cell-killing effect is known to decrease the radiation dose-related risk at high doses, because at a given level of dosage all cells die, even the cancerous ones, and thus, no secondary cancer could be seen in the organ. However, other issues could be observed, like the death of the organ. In our analysis, no cell-killing effect was demonstrated, such as that seen in other smaller organs such as the thyroid (Sigurdson et al. 2005).

Another childhood cancer survivors’ cohort, which did not include radiation dose reconstruction, estimated an SIR of 28.1 (95 % CI 9.1–65.7) and an AER of 28 per 100,000 person-years—values in accordance with our results (Cardous-Ubbink et al. 2007).

We estimated a higher increase in the risk of bone sarcoma following high radiation doses than that found in studies of retinoblastoma survivors (Kleinerman et al. 2005; Wong et al. 1997). This suggests that genetic retinoblastoma survivors, who have a much higher baseline risk than other cancer survivors due to a common genetic mechanism between retinoblastoma and sarcoma (Friend et al. 1986), do not exhibit greater sensitivity to radiation. A sensitivity analysis has been run excluding all retinoblastoma patients (results not shown); results were similar, so we kept them in our study to improve statistical power.

The new approach proposed in this article allows the evaluation of the radiation dose–response relationship in a cohort analysis, which exhibits a much greater precision than the usual case–control approach and allows for taking into account the radiation dose heterogeneity in the body. It should also avoid selection biases that may be found in case–control analyses.

## Conclusion

Based on a cohort of 4,171 survivors of childhood cancer with a median follow-up of 26 years and 39 incidents of second bone sarcomas, this study showed that the increase in the risk of bone sarcoma is well described by a linear function of the radiation dose received by the bones. Consequently, the risk of bone sarcoma is mainly a serious concern at high radiation doses. Also, it seems that almost all the risk of bone sarcoma is concentrated in the 30 years following childhood cancer treatment. However, it may be too early, based on the relatively short follow-up, to reach a conclusion on this aspect. Lastly, it appears that retinoblastoma, Ewing’s, soft tissue sarcomas and Hodgkin’s disease are primary cancer types with the highest risks of subsequent neoplasm. To conclude, it is crucial to lower the radiation dose to the bones because the risk of sarcoma increases continuously with increasing radiation dose to bones, without plateau. As much as is possible, other therapeutic solutions should be preferred to radiotherapy in bone sarcoma-sensitive areas, such as the head or pelvis. Also, intervention strategies such as screening or prevention of SMNs should be based on first cancer types with the highest risks of subsequent neoplasm.
